# Lateral Extra-Articular Tenodesis with Indirect Femoral Fixation Using an Anterior Cruciate Ligament Reconstruction Suspensory Device

**DOI:** 10.3390/jcm13020377

**Published:** 2024-01-10

**Authors:** Marco Bechis, Federica Rosso, Davide Blonna, Roberto Rossi, Davide Edoardo Bonasia

**Affiliations:** AO Ordine Mauriziano Hospital, Department of Orthopedics and Traumatology, University of Torino, 10124 Turin, Italy

**Keywords:** anterior cruciate ligament reconstruction, rotatory instability, iliotibial band, extra-articular tenodesis, extracortical suspensory device, complications, over-constraint, tunnel coalition

## Abstract

Background: The lateral extra-articular tenodesis (LET) procedure associated with anterior cruciate ligament (ACL) reconstruction can be considered in selected patients to diminish the risk of persistent rotatory instability and achieve a protective effect on the graft. Several techniques have been described in the literature to treat rotatory instability. Usually, a strip of the iliotibial band (ITB) is harvested from its middle while leaving the distal insertion, then passed underneath the lateral collateral ligament and fixed on the lateral aspect of the distal femur with various fixation methods such as staples, screws, anchors or extracortical suspensory devices. Despite their effectiveness, these fixation methods may be associated with complications such as lateral pain, over-constraint and tunnel convergence. Methods: This study presents a detailed surgical description of a new technique to perform an LET during ACL reconstruction with any type of graft fixing the ITB strip with the sutures of the ACL femoral button, comparing its pros and cons in relation to similar techniques found in the literature. Conclusions: This technique represents a reproducible, easy to learn and inexpensive solution to perform a lateral extra-articular tenodesis associated with an ACL reconstruction using the high-resistance sutures of the femoral button.

## 1. Introduction

Anterior cruciate ligament (ACL) injury is one of the most common sports-related injuries, with an incidence of 68.6 in 100,000 patients per year and affecting about 3% of amateur athletes each year [1], with percentages increasing by about five times when professional athletes are considered [2]. Several studies [3,4] demonstrated a higher prevalence of ACL rupture within the male population, with an almost twofold risk compared to the female population, despite the latter often being characterized by a higher presence of anatomical risk factors such as higher Q-angles, a smaller notch size and internal tibial rotation [5]. Other frequently cited risk factors for ACL rupture are generalized hyperlaxity, a steep tibial slope, high body-mass index and young age [6].

ACL injury can be managed with a non-surgical (rehabilitation) or surgical (reconstruction/reinsertion) approach. In the literature, a subgroup of patients, termed “coper”, is described to exhibit excellent movement control, improved balance, greater dynamic stability and fewer episodes of instability, along with satisfactory subjective and objective scores following a specialized course of neuromuscular and strength training. These individuals may, consequently, not require surgical intervention. Conversely, individuals termed “non-copers”, characterized by persistent instability, poor functional outcomes and subjective dissatisfaction despite undergoing appropriate physical therapy, tend to benefit more from a surgical approach [7]. Moreover, in recent years, there has been an increasing emergence of evidence regarding the importance of developing scores capable of identifying individuals at risk of injury and implementing specific preventive plyometrics, strength and neuromuscular programs aimed at reducing dynamic valgus, trunk lateralization and unequal limb loading [8].

It is generally accepted that a surgical approach is required for young and active patients to restore normal knee kinematics, to preserve the cartilage and menisci, as well as to increase the probability of returning to sports.

Over the past few decades, there have been major developments in surgical techniques, evolving from open, extra-articular and non-anatomical techniques to arthroscopic, intra-articular and anatomical techniques, including procedures such as double [9] or triple bundle [10] reconstructions or repairs with or without biological enhancement [11,12] and tibial spine avulsion reinsertion [13]. However, a significant percentage of patients (around 10–15% according to the literature [14]) reported unsatisfactory outcomes, with persistent anterior tibial translation and rotational instability. This can represent one of the causes of a reduced proportion of patients returning to sports as well as an increased risk of re-rupture or subsequent meniscal or cartilage injuries [15,16].

Antero-lateral rotatory instability (ALRI) is often associated with ACL injury, both acutely as a concomitant injury to the anterolateral complex and in chronic cases as the result of a progressive lengthening of the anterolateral structures due to the altered kinematics of the ACL-deficient knee [17]. In fact, different studies show that after isolated reconstruction of the ACL, the pivot shift phenomenon is present in 25–38% of patients [18], and this percentage drops significantly when an anterolateral procedure is associated [19]. Additionally, the re-rupture rate can reach values as high as 20% [16], particularly among young patients and elite athletes, and several studies [20,21] demonstrated that the incorporation of a lateral tenodesis can have a protective effect, reducing the risk by approximately 2.8 times without adversely affecting the return-to-sport rate and with a similar effect when combined with hamstring, quadriceps or patellar tendon autografts.

Over the years, different techniques have been described to address the anterolateral complex both in adult and pediatric patients. These techniques include using a strip of the iliotibial band (ITB) to be passed under the lateral collateral ligament (LCL) in different configurations and fixed at the level of the lateral femoral condyle [22,23,24,25,26] or sutured to itself [27], reconstruction of the anterolateral ligament (ALL) with an autograft [28] or allograft [29], direct repair of the ALL with an associated InternalBrace [30], or more complex techniques that allow concomitant reconstruction of the ACL and ALL (i.e., as described by Marcacci et al. [31], Yamaguchi et al. [32] and Saragaglia et al. [33]). The addition of a lateral extra-articular tenodesis could reduce the residual tibial anterior translation and the residual pivot shift, allowing for an earlier return to sports and providing better subjective outcomes. Many different variations of the LET approach have been introduced after Lemaire’s 1967 description of the original technique [26]. In one of the most frequently described variations, a 1 cm wide and 7–8 cm long strip of the ITB is harvested from its central portion preserving the distal insertion on the Gerdy’s tubercle. Then, it is passed below the LCL and fixed on the femur anterior and proximal to the lateral head of the gastrocnemius with staples, anchors or transosseous tunnels [34]. However, all these techniques are not free from complications, including the risk of over-constraining the lateral compartment [35] or convergence between ALL/LET femoral fixation and the ACL femoral tunnel [36]. This last complication should definitely be avoided when using extracortical fixation devices for ACL femoral fixation, since the device can be damaged by the suture anchors, staples or femoral tunnels used for anterolateral procedures.

In this paper, the authors describe step-by-step a new lateral extra-articular tenodesis technique using indirect femoral fixation, without any additional devices or femoral tunnels, with the goal of minimizing the risk of over-constraint and avoiding convergence with the ACL femoral tunnel.

## 2. Protocol

The technique described in the present paper can be performed when extracortical fixation devices are used for ACL femoral fixation (mostly when using the hamstrings or quadriceps tendon as a graft for ACLR). The authors’ indications for LET reflect the recent literature [37], and this technique is then performed if one or more of the following circumstances are present: (1) revision ACLR, (2) high-grade rotational laxity (grade-2 or -3 pivot shift), (3) generalized hyperlaxity or genu recurvatum of >10°, (4) young patients (<20 years old), (5) patients who aim to return to pivoting sports. Other secondary criteria that may be taken into account to determine the patient’s eligibility for this procedure are the presence of a Segond fracture, DLFNS (deep lateral femoral notch sign), obesity or total/subtotal lateral meniscectomy. There are no specific contraindications; however, caution should be observed in patients with a posterolateral corner injury and initial lateral compartment osteoarthritis [34].

### 2.1. Patient Positioning and Pre-Operative Evaluation

The patient is placed supine on the operating table, with a tourniquet located at the level of the proximal thigh and lateral support at this level to allow a knee hyperflexion up to 120°. Under general or spinal anesthesia, the stability of the knee is evaluated in all planes to confirm the ACL tear and to rule out any other ligamentous injuries. The following tests are used: Lachman test (positive if anterior tibial translation > 5 mm), anterior and posterior drawer test (positive if tibial translation > 5 mm anteriorly or posteriorly from a neutral starting position) [38], pivot shift test (positive if a glide or a clunk is observed) [38], varus-valgus stress maneuvers at 0° and 30° of flexion (normal values: no opening at 0° and <5° opening at 30° for both tests) [39].

### 2.2. Standard Arthroscopic Evaluation and ACL Reconstruction

After accurate disinfection with chlorexidine, standard anteromedial (AM) and anterolateral (AL) vertical arthroscopic portals are performed about 1 cm away from the lower pole of the patella. The cartilage status is evaluated, the cruciate ligaments’ tension is tested and the menisci are carefully screened to find any lesion needing specific treatment. The lateral meniscus is tested with the knee in figure-four position and under varus stress, while the medial meniscus is tested with the knee in extension and under valgus stress.

The authors perform the technique described mainly in association with soft tissue grafts (hamstrings or partial-thickness quadriceps tendon without bone block). While hamstrings are used in older and less active patients, the quadriceps tendon is preferred in young and active patients with a higher risk of re-rupture. Before implantation, the graft is pre-soaked in vancomycin (500 mg/100 mL) for 10 min and then prepared on a workstation.

The femoral tunnel is drilled using the anteromedial transportal technique aiming towards the anatomical femoral footprint and by using femoral offset guides (in order to preserve at least 2 mm of the posterior wall), while the tibial tunnel is drilled through the anteromedial surface of the tibia with the help of an angled ACL guide (55°–65°) inserted into the joint through the AM portal and with its tip positioned on the lateral wall of the medial tibial spine, in the center of the tibial stump.

With the help of a shuttle suture, the graft is passed into the tibial tunnel first and then into the femoral tunnel by gentle traction. After the femoral fixation, the knee is cycled several times, checking the full range of motion and ruling out graft impingement. The femoral fixation is achieved with an adjustable extracortical suspensory device regardless of the graft type, be it the quadriceps tendon or hamstrings; in the instance of the quadriceps tendon, the tibial fixation is performed in full extension with a 20 mm diameter round button attached to an adjustable loop and secured with at least three simple knots, whereas for the hamstrings the graft is secured at 20° of flexion with a bioabsorbable interference screw (diameter of the screw 1 mm larger than the tibial tunnel).

### 2.3. Lateral Extra-Articular Tenodesis

The following procedure is always performed after an ACL reconstruction.

**Figure 1:**
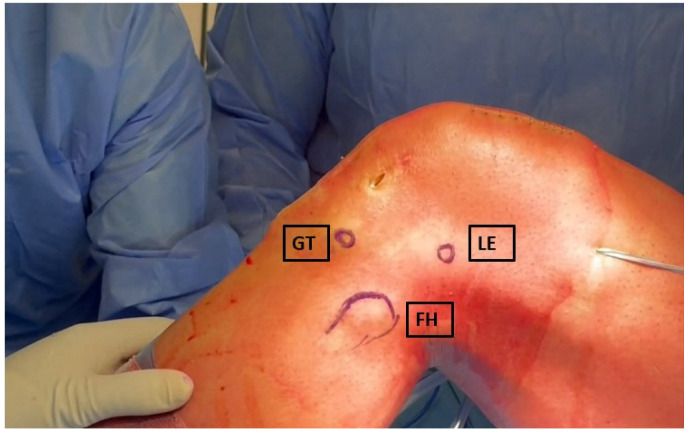
Skin landmarks. GT: Gerdy’s tubercle, LE: lateral epicondyle, FH: fibular head.

With the knee at 90° of flexion, the fibular head, lateral epicondyle and Gerdy’s tubercle are identified by palpation and marked. A straight 5 cm longitudinal incision centered on the lateral femoral epicondyle is then performed (Figure 1).

**Figure 2:**
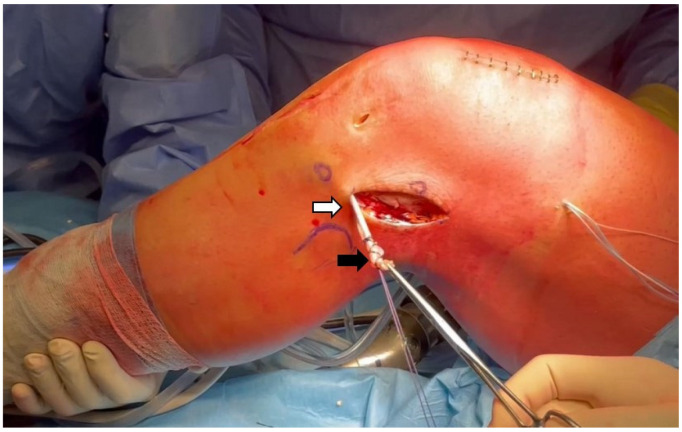
ITB strip harvesting and preparation. Harvesting of a strip from the central third of the ITB (8 × 1 cm) (white arrow). Its distal insertion is preserved while the proximal part is armed with a number-2 multifilament adsorbable suture (black arrow).

The subcutaneous fat is dissected with blunt scissors down to the iliotibial band (ITB). An 8 cm long, 1 cm wide strip of the ITB is harvested, preserving the distal attachment and centering the incisions on the lateral femoral epicondyle in both the anterior–posterior and proximal–distal directions. A whipstitch with a number-2 multifilament adsorbable suture is placed in the proximal free end of the strip for 3 cm (Figure 2).

**Figure 3:**
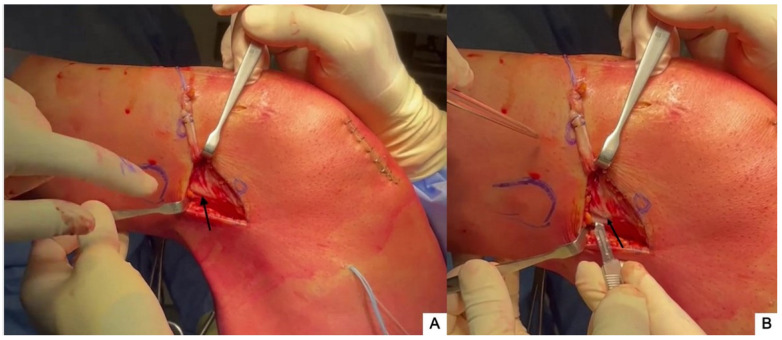
(**A**) The LCL (black arrow) is identified by palpation with the knee in a figure-4 position. (**B**) A 1 cm incision is then performed posterior and parallel to the LCL (black arrow).

The lateral collateral ligament (LCL) is identified by palpation (and by placing the knee in a figure-four position) (Figure 3A) and the soft tissues posterior to the LCL are incised parallel to it (~1 cm long).

**Figure 4:**
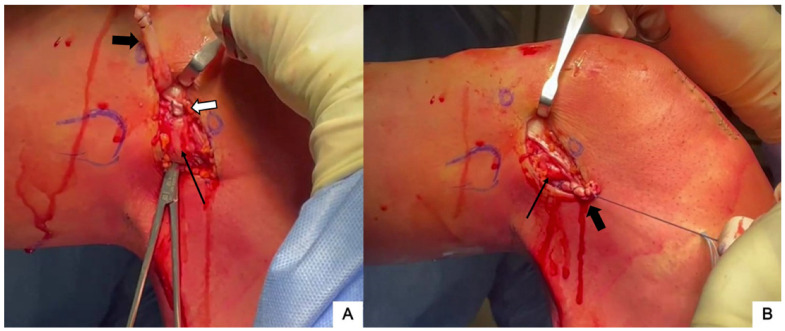
A 90° suture passer is advanced deep into the LCL (thin arrow) and the tip (white arrow) is directed anteriorly to grasp the arming sutures of the ITB strip (**A**) and retrieve the graft (black arrow) under the LCL (**B**).

A 90° suture passer is advanced deep into the LCL and directed anteriorly until it can be palpated. A second 1 cm sharp incision is made parallel to the LCL at the tip of the suture passer. The suture passer is then used to retrieve the polyglactin arming sutures deep in the LCL through the proximal incision (Figure 4A).

The ITB strip is driven through the same path by gently pulling on the arming sutures and by cycling the knee (Figure 4B).

**Figure 5:**
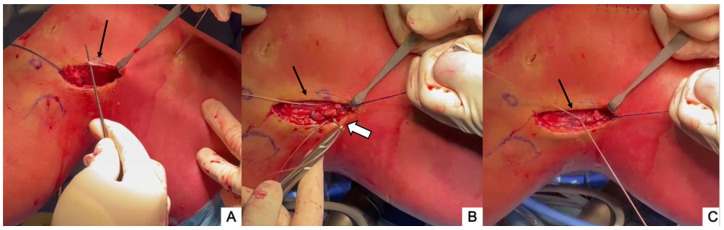
With the help of a probe inserted under the ITB, the high-resistance sutures (thin black arrow) of the adjustable loop are retrieved (**A**) and a free needle is used to pass them through the proximal part of the ITB strip (white arrow) (**B**). Final fixation is then performed with the knee at 30° of flexion and neutral rotation (**C**).

The high-resistance sutures of the adjustable loop are retrieved under the vastus lateralis and through the ITB incision by inserting a finger or a probe, while keeping some tension on the adjustable loop sutures. (Figure 5A). A free needle is used to pass the high-resistance sutures through the proximal part of the ITB strip (Figure 5B) and to place two to three simple interrupted sutures secured with at least three knots. The final fixation is performed with the knee at 30° of flexion and the foot in neutral rotation (Figure 5C).

After wound irrigation and final hemostasis, the iliotibial band is completely closed with a number-2 polyglactin continuous locking suture (Supplementary Video S1).

### 2.4. Post-Operative Protocol

The procedure takes approximately 10–15 min to complete, with the tourniquet being released just before the skin closure for a final check for bleeding.

**Figure 6:**
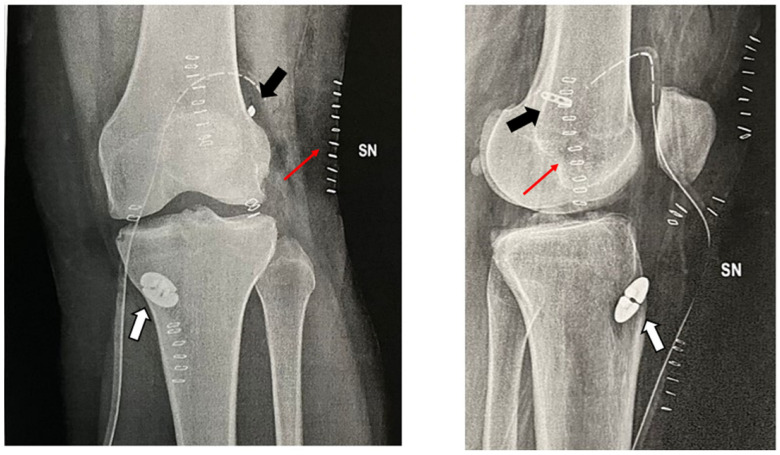
Post-operative X-rays. The surgical drainage is still in place, and the graft is fixed with two adjustable loop suspensory devices (black arrow) and a dedicated button on the tibial side (white arrow). On the lateral projection, only the skin staples (thin red arrow) can be appreciated at the level of the ITB harvesting site, since no additional hardware was used to fix the ITB strip.

Post-operatively, full-weight-bearing as tolerated and immediate ROM and muscle-strengthening exercises are allowed from the first post-operative day. The addition of the lateral tenodesis does not alter in any way the standard ACL reconstruction post-operative regimen, weight-bearing allowance or the type of physiotherapy protocol, all of which are predominantly influenced by additional procedures at the meniscal level (e.g., selective meniscectomy, sutures, root reinsertion) or at the cartilage level (e.g., microfractures, OATS, AMIC)**.** Post-operative X-rays are shown (Figure 6).

## 3. Discussion

The persistence of rotational instability after ACL reconstruction can be as high as 25% of cases, resulting in poor outcomes and an increased risk of re-rupture [40,41]. The modified Lemaire LET has recently been shown to reduce antero-lateral rotatory laxity in knees after ACL reconstruction [19,42] and, as described by Geeslin et al. [43], the association of ACL reconstruction with an ALL reconstruction or LET could also reduce the residual anterior tibial translation. Other studies have shown that the addition of an extra-articular procedure to ACL reconstruction significantly reduced the prevalence of residual pivot shift, allowing for an earlier return to sports and providing better subjective outcomes [44,45,46]. Furthermore, several studies have demonstrated its effectiveness in reducing the re-rupture rate, both when coupled with ACL reconstructions using hamstring [47] or bone–patellar tendon–bone grafts [48]. Several studies with long-term follow-ups ranging from 20 to 25 years demonstrate high patient-satisfaction rates [49,50]. Moreover, combining a lateral extra-articular tenodesis with anterior cruciate ligament reconstruction has shown long-term protective effects in reducing the risk of arthritis compared to isolated ACL reconstruction [51].

Numerous variations of the isolated LET are described in the literature. In particular, the main differences concern proximal fixation, which can be performed using suture anchors [52], interference screws [53], extracortical suspensory systems [54], staples [55] or by suturing the ITB graft onto itself [56]. Each of these fixation methods has proven effective, with no differences reported in the literature regarding failure rates and patient-reported outcomes; however, different potential risks and complications are described. An additional transosseous tunnel at the femoral level may introduce a risk of convergence with other tunnels [36], especially in cases of revision ACL or multiligament reconstructions. Metallic staples can cause irritative lateral pain, pose a risk of damaging the ACL tunnel and may undergo mobilization [57]—a phenomenon that can also occur with suture anchors or interference screws. In the literature, there is limited clinical and biomechanical evidence of the superiority of one fixation method over another, with only one study conducted by Behrendt et al. [58] directly comparing onlay anchor fixation and transosseous interference screw fixation, which did not find any differences in terms of Tegner score and anterior tibial translation. Our modification utilizes an indirect femoral fixation method employing the high-resistance sutures of the ACL-reconstruction femoral extracortical fixation device. This simple, rapid, cost-effective approach is currently the authors’ favorite technique for combined LET and ACLR procedures, when a proximal suspensory fixation is used. Although within our clinical practice this technique is almost exclusively used if femoral fixation is carried out with suspensory fixation devices, it can also be extended to other types of grafts. When performing bone–patellar tendon–bone (BPTB) ACLR, the graft is usually fixed on the femur with an interference screw. In this case, there is no risk of damaging the suspensory fixation device when performing a combined LET and any fixation device (staples, suture anchors) can be used for ITB strip fixation. However, the technique described in this article can be used also with BPTB: in this case, the arming sutures used to pull the bone plug into the femoral tunnel can be used to fix the LET. Alternatively, the BPTB graft can be fixed on the femur with dedicated suspensory fixation devices and is no different from other grafts fixed in the same fashion.

In addition to being inexpensive with no extra fixation devices, this technique provides two other important advantages: (1) one can avoid graft over-tensioning and (2) there is no need for a LET femoral bone tunnel. In fact, ACL reconstruction can prevent secondary joint damage and halt post-traumatic osteoarthritis of the knee [59]. However, with the addition of LET, concerns exist regarding the restriction of external rotation, pain and subsequent lateral over-constraint [60]. Since this technique does not entail rigid fixation and provides the possibility of manually adjusting the tension, it could minimize the risk of over-constraining the lateral compartment. According to current biomechanical research, a significantly low tension of 20 N is adequate to prevent over-constraint and restore normal knee kinematics [61].

Some studies have also noted that proper intraoperative planning is necessary to avoid dangerous convergence between the femoral tunnels in combined ACL reconstruction and LET [36,62]. This is also a major issue not only in primary ACL reconstructions, but especially in revision ACL reconstruction, where tunnel management is a crucial aspect of the preoperative planning. Finally, with this technique there is no need for additional hardware, an advantage that is especially useful against metal hardware that can have complications such as painful lateral irritation, resulting in the need for their removal.

The combination of all these features is what makes this technique unique and worthy of consideration. To the best of our knowledge, one study [63] in the literature described a similar technique in pediatric all-inside partial epiphyseal ACLR, while two other studies performed a modified lateral tenodesis in adult patients by fixing the ITB graft onto itself using high-resistance sutures [56] or by interposing it between the bone and the femoral button [64]. In our experience, this technique can be used in adult patients as well without major complications; moreover, it can be associated with an ACL reconstruction performed using the standard transportal technique, thus not requiring additional instruments as in the case of the all-inside or out–in technique. Suturing the ITB graft onto itself theoretically poses a risk, especially in thin patients or patients with low muscle mass, of creating lateral prominence with associated discomfort. Additionally, fixation to the femoral button allows, as also highlighted by Koukoulias et al. [64], a hypothetically greater integration and healing of the ITB graft due to its proximity to the femoral tunnel and the associated bleeding under it. The technique described by Koukoulias et al. [52] appears, therefore, very similar to ours from a functional and anatomical standpoint; however, we believe that the fixation of the ACL graft and the LET should be performed independently in order to achieve a secure embedding of the femoral button onto the lateral femoral cortex and avoid issues related to simultaneous tensioning of the two grafts, which could compromise their stability.

Some limitations of this technique are the possible difficulty in retrieving the high-resistance sutures of the adjustable loop through the ITB incision, which can be time-consuming, and the need for biomechanical validation when compared to other techniques. Since the technique involves an indirect femoral fixation, there is also a theoretical risk of not providing any protection to the graft if the knot becomes loose; however, if the ITB strip is fixed with two to three simple interrupted sutures secured with at least three knots this is an extremely rare occurrence, and thus far, we have had no failure in our clinical practice. Since this is a technique that has recently been introduced into our clinical practice (less than two years), unfortunately we do not yet have a sufficient number of patients with adequate follow-up to present statistically significant data regarding its safety, long-term outcome data or comparison with other existing techniques. However, extracortical suspensory devices are widely employed for ligament reconstructions with optimal biomechanical strength, and in our technique the femoral button mimics the function of a suture anchor, where the high-resistance sutures are used to fix the ITB graft. Furthermore, as highlighted in a prior study [64], proximity to the femoral tunnel may promote enhanced integration of the ITB graft due to the presence of healing factors associated with bleeding through the femoral tunnel itself. Based on our current provisional and unpublished data on almost one-hundred patients, recurrent instability, re-tear and complications specifically related to the described procedure (hematoma, lateral irritative pain, skin complications, infection) were not observed in our series. Table 1 presents a comprehensive review of the main advantages and disadvantages of this technique.

## 4. Conclusions

This technique represents a reproducible, easy to learn and inexpensive solution to perform a lateral extra-articular tenodesis associated with an ACL reconstruction using the high-resistance sutures of the femoral button, without the need of any other implants (staples, anchors or screws) and reducing the risk of tunnel coalition. The technique allows for independent tensioning of the ACL graft and the ITB strip, achieving a secure indirect femoral fixation of the latter near the femoral tunnel, where the bleeding under it might promote graft healing. Further future studies are needed to validate its effectiveness from a biomechanical point of view, to compare it with other existing techniques and to investigate its medium- to long-term outcomes.

## Figures and Tables

**Table 1:** This table summarizes advantages and limitations of the technique.

Advantages	Disadvantages/Limitations
Easy, reproducible and cost-effective	Potential risk of knots loosening
Can be used safely both in adult and pediatric patients	Retrieving sutures under vastus lateral may initially be challenging
Applicable with all type of ACL grafts	The biomechanical properties of this fixation technique and direct comparisons with already-existing techniques have not yet been studied
No additional devices needed	Lack of long-term objective and subjective outcomes
No additional femoral bone tunnel	
Proximity to femoral bone tunnel and potential graft-enhanced integration due to bleeding under it	
Independent fixation of ACL graft and ITB strip	

## Data Availability

Data are contained within the article and Supplementary Materials.

## Supplementary Materials

The following supporting information can be downloaded at: https://www.mdpi.com/article/10.3390/jcm13020377/s1.

## References

[1] Sanders T.L., Maradit Kremers H., Bryan A.J., Larson D.R., Dahm D.L., Levy B.A., Stuart M.J., Krych A.J. (2016). Incidence of Anterior Cruciate Ligament Tears and Reconstruction: A 21-Year Population-Based Study. Am. J. Sports Med..

[2] Costa G.G., Perelli S., Grassi A., Russo A., Zaffagnini S., Monllau J.C. (2022). Minimizing the Risk of Graft Failure after Anterior Cruciate Ligament Reconstruction in Athletes. A Narrative Review of the Current Evidence. J. Exp. Orthop..

[3] Nordenvall R., Bahmanyar S., Adami J., Stenros C., Wredmark T., Felländer-Tsai L. (2012). A Population-Based Nationwide Study of Cruciate Ligament Injury in Sweden, 2001-2009: Incidence, Treatment, and Sex Differences. Am. J. Sports Med..

[4] Sayampanathan A.A., Howe B.K.T., Bin Abd Razak H.R., Chi C.H., Tan A.H.C. (2017). Epidemiology of Surgically Managed Anterior Cruciate Ligament Ruptures in a Sports Surgery Practice. J. Orthop. Surg..

[5] Laible C., Sherman O.H. (2014). Risk Factors and Prevention Strategies of Non-Contact Anterior Cruciate Ligament Injuries. Bull. NYU Hosp. Jt. Dis..

[6] Smith H.C., Vacek P., Johnson R.J., Slauterbeck J.R., Hashemi J., Shultz S., Beynnon B.D. (2012). Risk Factors for Anterior Cruciate Ligament Injury: A Review of the Literature—Part 1: Neuromuscular and Anatomic Risk. Sports Health.

[7] Thoma L.M., Grindem H., Logerstedt D., Axe M., Engebretsen L., Risberg M.A., Snyder-Mackler L. (2019). Coper Classification Early After Anterior Cruciate Ligament Rupture Changes With Progressive Neuromuscular and Strength Training and Is Associated With 2-Year Success: The Delaware-Oslo ACL Cohort Study. Am. J. Sports Med..

[8] Nessler T., Denney L., Sampley J. (2017). ACL Injury Prevention: What Does Research Tell Us?. Curr. Rev. Musculoskelet. Med..

[9] Seppänen A., Suomalainen P., Huhtala H., Mäenpää H., Kiekara T., Järvelä T. (2021). Double Bundle ACL Reconstruction Leads to Better Restoration of Knee Laxity and Subjective Outcomes than Single Bundle ACL Reconstruction. Knee Surg. Sports Traumatol. Arthrosc..

[10] Uchida R., Shino K., Iuchi R., Tachibana Y., Yokoi H., Nakagawa S., Mae T. (2021). Anatomical Triple Bundle Anterior Cruciate Ligament Reconstructions With Hamstring Tendon Autografts: Tunnel Locations and 2-Year Clinical Outcomes. Arthrosc. J. Arthrosc. Relat. Surg..

[11] Murray M.M., Fleming B.C., Badger G.J., Freiberger C., Henderson R., Barnett S., Kiapour A., Ecklund K., Proffen B., Sant N. (2020). Bridge-Enhanced Anterior Cruciate Ligament Repair Is Not Inferior to Autograft Anterior Cruciate Ligament Reconstruction at 2 Years: Results of a Prospective Randomized Clinical Trial. Am. J. Sports Med..

[12] Taylor S.A., Khair M.M., Roberts T.R., Difelice G.S. (2015). Primary Repair of the Anterior Cruciate Ligament: A Systematic Review. Arthrosc. J. Arthrosc. Relat. Surg..

[13] Russu O.M., Pop T.S., Ciorcila E., Gergely I., Zuh S.-G., Trâmbițaș C., Borodi P.G., Incze-Bartha Z., Feier A.M., Georgeanu V.A. (2021). Arthroscopic Repair in Tibial Spine Avulsion Fractures Using Polyethylene Terephthalate Suture: Good to Excellent Results in Pediatric Patients. J. Pers. Med..

[14] Samitier G., Marcano A.I., Alentorn-Geli E., Cugat R., Farmer K.W., Moser M.W. (2015). Failure of Anterior Cruciate Ligament Reconstruction. Arch. Bone Jt. Surg..

[15] Ardern C.L., Taylor N.F., Feller J.A., Webster K.E. (2014). Fifty-Five per Cent Return to Competitive Sport Following Anterior Cruciate Ligament Reconstruction Surgery: An Updated Systematic Review and Meta-Analysis Including Aspects of Physical Functioning and Contextual Factors. Br. J. Sports Med..

[16] Lai C.C.H., Ardern C.L., Feller J.A., Webster K.E. (2018). Eighty-Three per Cent of Elite Athletes Return to Preinjury Sport after Anterior Cruciate Ligament Reconstruction: A Systematic Review with Meta-Analysis of Return to Sport Rates, Graft Rupture Rates and Performance Outcomes. Br. J. Sports Med..

[17] Ellison A.E. (1980). The Pathogenesis and Treatment of Anterolateral Rotatory Instability. Clin. Orthop. Relat. Res..

[18] Dodds A.L., Gupte C.M., Neyret P., Williams A.M., Amis A.A. (2011). Extra-Articular Techniques in Anterior Cruciate Ligament Reconstruction: A Literature Review. J. Bone Jt. Surg. Vol..

[19] Hewison C.E., Tran M.N., Kaniki N., Remtulla A., Bryant D., Getgood A.M. (2015). Lateral Extra-Articular Tenodesis Reduces Rotational Laxity When Combined with Anterior Cruciate Ligament Reconstruction: A Systematic Review of the Literature. Arthrosc. J. Arthrosc. Relat. Surg..

[20] Noyes F.R., Barber S.D. (1991). The Effect of an Extra-Articular Procedure on Allograft Reconstructions for Chronic Ruptures of the Anterior Cruciate Ligament. J. Bone Jt. Surg..

[21] Borque K.A., Jones M., Laughlin M.S., Balendra G., Willinger L., Pinheiro V.H., Williams A. (2022). Effect of Lateral Extra-Articular Tenodesis on the Rate of Revision Anterior Cruciate Ligament Reconstruction in Elite Athletes. Am. J. Sports Med..

[22] Lagae K.C., Robberecht J., Athwal K.K., Verdonk P.C.M., Amis A.A. (2020). ACL Reconstruction Combined with Lateral Monoloop Tenodesis Can Restore Intact Knee Laxity. Knee Surg. Sports Traumatol. Arthrosc..

[23] Christel P., Djian P. (2002). [Anterio-Lateral Extra-Articular Tenodesis of the Knee Using a Short Strip of Fascia Lata]. Rev. Chir. Orthop. Reparatrice L’appareil Mot..

[24] Andrews J.R., Sanders R. (1983). A “mini-Reconstruction” Technique in Treating Anterolateral Rotatory Instability (ALRI). Clin. Orthop. Relat. Res..

[25] Losee R.E., Johnson T.R., Southwick W.O. (1978). Anterior Subluxation of the Lateral Tibial Plateau. A Diagnostic Test and Operative Repair. J. Bone Jt. Surg..

[26] Lemaire M. (1967). Rupture Ancienne Du Ligament Croisé Antérieur Du Genou. J. Chir..

[27] Arnold J.A., Coker T.P., Heaton L.M., Park J.P., Harris W.D. (1979). Natural History of Anterior Cruciate Tears. Am. J. Sports Med..

[28] Sonnery-Cottet B., Thaunat M., Freychet B., Pupim B.H.B., Murphy C.G., Claes S. (2015). Outcome of a Combined Anterior Cruciate Ligament and Anterolateral Ligament Reconstruction Technique with a Minimum 2-Year Follow-Up. Am. J. Sports Med..

[29] Chahla J., Menge T.J., Mitchell J.J., Dean C.S., LaPrade R.F. (2016). Anterolateral Ligament Reconstruction Technique: An Anatomic-Based Approach. Arthrosc. Tech..

[30] Hopper G.P., Aithie J.M.S., Jenkins J.M., Wilson W.T., Mackay G.M. (2020). Combined Anterior Cruciate Ligament Repair and Anterolateral Ligament Internal Brace Augmentation: Minimum 2-Year Patient-Reported Outcome Measures. Orthop. J. Sports Med..

[31] Marcacci M., Zaffagnini S., Marcheggiani Muccioli G.M., Neri M.P., Bondi A., Nitri M., Bonanzinga T., Grassi A. (2011). Arthroscopic Intra- and Extra-Articular Anterior Cruciate Ligament Reconstruction with Gracilis and Semitendinosus Tendons: A Review. Curr. Rev. Musculoskelet. Med..

[32] Yamaguchi S., Sasho T., Tsuchiya A., Wada Y., Moriya H. (2006). Long Term Results of Anterior Cruciate Ligament Reconstruction with Iliotibial Tract: 6-, 13-, and 24-Year Longitudinal Follow-Up. Knee Surg. Sports Traumatol. Arthrosc..

[33] Saragaglia D., Pison A., Refaie R. (2013). Lateral Tenodesis Combined with Anterior Cruciate Ligament Reconstruction Using a Unique Semitendinosus and Gracilis Transplant. Int. Orthop..

[34] Jesani S., Getgood A. (2019). Modified Lemaire Lateral Extra-Articular Tenodesis Augmentation of Anterior Cruciate Ligament Reconstruction. JBJS Essent. Surg. Tech..

[35] Devitt B.M., Bouguennec N., Barfod K.W., Porter T., Webster K.E., Feller J.A. (2017). Combined Anterior Cruciate Ligament Reconstruction and Lateral Extra-Articular Tenodesis Does Not Result in an Increased Rate of Osteoarthritis: A Systematic Review and Best Evidence Synthesis. Knee Surg. Sports Traumatol. Arthrosc..

[36] Jaecker V., Ibe P., Endler C.H., Pfeiffer T.R., Herbort M., Shafizadeh S. (2019). High Risk of Tunnel Convergence in Combined Anterior Cruciate Ligament Reconstruction and Lateral Extra-Articular Tenodesis. Am. J. Sports Med..

[37] Sonnery-Cottet B., Daggett M., Fayard J.M., Ferretti A., Helito C.P., Lind M., Monaco E., de Pádua V.B.C., Thaunat M., Wilson A. (2017). Anterolateral Ligament Expert Group Consensus Paper on the Management of Internal Rotation and Instability of the Anterior Cruciate Ligament-Deficient Knee. J. Orthop. Traumatol..

[38] Van Eck C.F., van Den Bekerom M.P., Fu F.H., Poolman R.W., Kerkhoffs G.M. (2013). Methods to Diagnose Acute Anterior Cruciate Ligament Rupture: A Meta-Analysis of Physical Examinations with and without Anaesthesia. Knee Surg. Sports Traumatol. Arthrosc..

[39] Lubowitz J.H., Bernardini B.J., Reid J.B. (2008). Current Concepts Review: Comprehensive Physical Examination for Instability of the Knee. Am. J. Sports Med..

[40] Chouliaras V., Ristanis S., Moraiti C., Stergiou N., Georgoulis A.D. (2007). Effectiveness of Reconstruction of the Anterior Cruciate Ligament with Quadrupled Hamstrings and Bone-Patellar Tendon-Bone Autografts: An in Vivo Study Comparing Tibial Internal-External Rotation. Am. J. Sports Med..

[41] Lohmander L.S., Englund P.M., Dahl L.L., Roos E.M. (2007). The Long-Term Consequence of Anterior Cruciate Ligament and Meniscus Injuries: Osteoarthritis. Am. J. Sports Med..

[42] Getgood A.M., Bryant D.M., Litchfield R., Heard M., McCormack R.G., Rezansoff A., Peterson D., Bardana D., MacDonald P.B., Verdonk P.C. (2020). Lateral Extra-Articular Tenodesis Reduces Failure of Hamstring Tendon Autograft Anterior Cruciate Ligament Reconstruction: 2-Year Outcomes From the STABILITY Study Randomized Clinical Trial. Am. J. Sports Med..

[43] Geeslin A.G., Moatshe G., Chahla J., Kruckeberg B.M., Muckenhirn K.J., Dornan G.J., Coggins A., Brady A.W., Getgood A.M., Godin J.A. (2018). Anterolateral Knee Extra-Articular Stabilizers: A Robotic Study Comparing Anterolateral Ligament Reconstruction and Modified Lemaire Lateral Extra-Articular Tenodesis. Am. J. Sports Med..

[44] Vadalà A.P., Iorio R., De Carli A., Bonifazi A., Iorio C., Gatti A., Rossi C., Ferretti A. (2013). An Extra-Articular Procedure Improves the Clinical Outcome in Anterior Cruciate Ligament Reconstruction with Hamstrings in Female Athletes. Int. Orthop..

[45] Zaffagnini S., Marcacci M., Lo Presti M., Giordano G., Iacono F., Neri M.P. (2006). Prospective and Randomized Evaluation of ACL Reconstruction with Three Techniques: A Clinical and Radiographic Evaluation at 5 Years Follow-Up. Knee Surg. Sports Traumatol. Arthrosc..

[46] Rezende F.C., de Moraes V.Y., Martimbianco A.L.C., Luzo M.V., da Silveira Franciozi C.E., Belloti J.C. (2015). Does Combined Intra- and Extraarticular ACL Reconstruction Improve Function and Stability? A Meta-Analysis. Clin. Orthop. Relat. Res..

[47] Porter M., Shadbolt B. (2020). Modified Iliotibial Band Tenodesis Is Indicated to Correct Intraoperative Residual Pivot Shift After Anterior Cruciate Ligament Reconstruction Using an Autologous Hamstring Tendon Graft: A Prospective Randomized Controlled Trial. Am. J. Sports Med..

[48] Castoldi M., Magnussen R.A., Gunst S., Batailler C., Neyret P., Lustig S., Servien E. (2020). A Randomized Controlled Trial of Bone-Patellar Tendon-Bone Anterior Cruciate Ligament Reconstruction With and Without Lateral Extra-Articular Tenodesis: 19-Year Clinical and Radiological Follow-up. Am. J. Sports Med..

[49] Zaffagnini S., Marcheggiani Muccioli G.M., Grassi A., Roberti Di Sarsina T., Raggi F., Signorelli C., Urrizola F., Spinnato P., Rimondi E., Marcacci M. (2017). Over-the-Top ACL Reconstruction Plus Extra-Articular Lateral Tenodesis With Hamstring Tendon Grafts: Prospective Evaluation With 20-Year Minimum Follow-up. Am. J. Sports Med..

[50] Pernin J., Verdonk P., Si Selmi T.A., Massin P., Neyret P. (2010). Long-term follow-up of 24.5 years after intra-articular anterior cruciate ligament reconstruction with lateral extra-articular augmentation. Am. J. Sports Med..

[51] Viglietta E., Ponzo A., Monaco E., Iorio R., Drogo P., Andreozzi V., Conteduca F., Ferretti A. (2022). ACL Reconstruction Combined With the Arnold-Coker Modification of the MacIntosh Lateral Extra-Articular Tenodesis: Long-Term Clinical and Radiological Outcomes. Am. J. Sports Med..

[52] Muller B., Willinge G.J.A., Zijl J.A.C. (2020). Minimally Invasive Modified Lemaire Tenodesis. Arthrosc. Tech..

[53] Puzzitiello R.N., Agarwalla A., Bush-Joseph C.A., Forsythe B. (2019). Iliotibial Band Tenodesis With a Tenodesis Screw for Augmentation of Anterior Cruciate Ligament Reconstruction. Arthrosc. Tech..

[54] Singh S., Shaunak S., Shaw S.C.K., Anderson J.L., Mandalia V. (2020). Adjustable Loop Femoral Cortical Suspension Devices for Anterior Cruciate Ligament Reconstruction: A Systematic Review. Indian J. Orthop..

[55] Kwapisz A., Mollison S., McRae S., MacDonald P. (2019). Lateral Extra-Articular Tenodesis With Proximal Staple Fixation. Arthrosc. Tech..

[56] Abusleme S., Strömbäck L., Caracciolo G., Zamorano H., Cheyre J., Vergara F., Yañez R. (2021). Lateral Extra-Articular Tenodesis: A Technique With an Iliotibial Band Strand Without Implants. Arthrosc. Tech..

[57] Moran T.E., MacLean I.S., Anderson G.R., Barras L.A., Graf R.M., Diduch D.R., Miller M.D. (2022). Lateral Extra-Articular Tenodesis Staple Risks Penetration of Anterior Cruciate Ligament Reconstruction Tunnel. Arthrosc. Sports Med. Rehabil..

[58] Behrendt P., Fahlbusch H., Akoto R., Thürig G., Frings J., Herbst E., Raschke M.J., Frosch K.H., Kittl C., Krause M. (2023). Comparison of Onlay Anchor Fixation Versus Transosseous Fixation for Lateral Extra-Articular Tenodesis During Revision ACL Reconstruction. Orthop. J. Sports Med..

[59] Cheung E.C., DiLallo M., Feeley B.T., Lansdown D.A. (2020). Osteoarthritis and ACL Reconstruction-Myths and Risks. Curr. Rev. Musculoskelet. Med..

[60] Neri T., Cadman J., Beach A., Grasso S., Dabirrahmani D., Putnis S., Oshima T., Devitt B., Coolican M., Fritsch B. (2021). Lateral Tenodesis Procedures Increase Lateral Compartment Pressures More than Anterolateral Ligament Reconstruction, When Performed in Combination with ACL Reconstruction: A Pilot Biomechanical Study. J. ISAKOS.

[61] Inderhaug E., Stephen J.M., El-Daou H., Williams A., Amis A.A. (2017). The Effects of Anterolateral Tenodesis on Tibiofemoral Contact Pressures and Kinematics. Am. J. Sports Med..

[62] Kittl C., Schwietering L., Raschke M.J., Frank A., Glasbrenner J., Wagner M., Herbort M., Weiler A. (2022). Tunnel Convergence Rate in Combined Anteromedial Portal Anterior Cruciate Ligament and Anterolateral Structure Reconstructions Is Influenced by Anterior Cruciate Ligament Knee Flexion Angle, Tunnel Position, and Direction. Arthrosc. J. Arthrosc. Relat. Surg..

[63] Leyes-Vence M., Roca-Sanchez T., Flores-Lozano C., Villarreal-Villareal G. (2019). All-Inside Partial Epiphyseal Anterior Cruciate Ligament Reconstruction Plus an Associated Modified Lemaire Procedure Sutured to the Femoral Button. Arthrosc. Tech..

[64] Koukoulias N.E., Dimitriadis T., Vasiliadis A.V., Germanou E., Boutovinos A.P. (2022). ACL Reconstruction and Modified Lemaire Tenodesis Utilizing Common Suspensory Femoral Fixation. Arthrosc. Tech..

